# All optical modulation in vertically coupled indium tin oxide ring resonator employing epsilon near zero state

**DOI:** 10.1038/s41598-023-44438-3

**Published:** 2023-10-26

**Authors:** Swati Rajput, Vishal Kaushik, Prem Babu, Suresh K. Pandey, Mukesh Kumar

**Affiliations:** 1 Department of Electrical Engineering, Indian Institute of Technology (IIT) Jodhpur, Jodhpur, India; 2 School of Electrical Engineering, Tel Aviv University, Tel Aviv, Israel; 3 Department of Electrical Engineering, Indian Institute of Technology (IIT) Indore, Indore, India; 4 Center of Advanced Electronics, Indian Institute of Technology (IIT) Indore, Indore, India

**Keywords:** Microresonators, Silicon photonics, Ultrafast photonics, Optoelectronic devices and components, Optics and photonics, Nanophotonics and plasmonics

## Abstract

We present an innovative approach to achieve all-optical modulation within an ITO-based vertically coupled ring resonator. This method leverages the material's enhanced nonlinear response in the near-infrared wavelengths, particularly within the epsilon-near-zero (ENZ) state. To enhance the interaction between light and the material while minimizing scattering losses, our approach employs an ITO-based vertically connected ring resonator. The vertical arrangement eliminates the need for etching fine gaps to separate the ring and bus waveguide. The novel waveguide design addresses the necessity of high sensitivity, non-linear effects and compact size opening the possibilities for all-optical signal processing. This unique resonator structure effectively facilitates the coupling of a high-intensity pump wavelength into the ITO-based micro-ring resonator. Consequently, this optical pumping induces electron heating within the ITO material, leading to a significant increase in its nonlinear optical properties. This, in turn, results in a noteworthy alteration of ITO's refractive index, specifically in the unity order, thereby modifying the complex effective index of the optical beam propagating at 1550 nm. Our experimental findings demonstrate an impressive extinction ratio of 18 dB for a 30 µm long device, which highlights the efficiency of our approach in achieving all-optical modulation through the optical pumping of an ITO-based vertically coupled ring resonator. The proposed all-optical modulator has outperformed as compared to conventional waveguide-based modulators in terms of extinction ratio and footprint. This novel technique holds immense potential for advancing high-speed data communication systems in the future. As the demand for advanced processing capabilities, such as artificial intelligence, continues to grow, all-optical modulation emerges as a groundbreaking technology poised to revolutionize the next generation of computing and communication systems.

## Introduction

Empowering Data at the Speed of Light: Optical Modulators Illuminate the Future. The greatest potential and assurance of photonics is to counteract the inherent limitations of electronics, including bandwidth and device heating. Numerous notable advances in this area focus solely over the use of light like an information carrier, laying the groundwork for light-based systems that will have a significant influence of lowered energy usage and competent efficiency^1–4^. The most popular method for providing active control of light is electro-optical modulators^5,6^. Indeed, the performance of pure Silicon-based optical modulators has increased significantly in recent years, most notably the bandwidth, which has now approached the GHz range. But since we clearly know that optical networks have unending demands, it is still uncertain whether Si alone can achieve the necessary performance parameters. The weak electro-optic effect, unstable thermo-optical effect, huge device size, and significant propagation losses are still problems for pure Si-based modulators or hybrid Si-based optical modulators including plasmonic or another electro-optic material. Despite being used in industry, they have constrained bandwidth and high-power consumption due to the significant electronics involved^7–9^. To achieve a highly efficient and fully customizable control of optical states at scales much below the diffraction limit of electromagnetic radiation, novel, inexpensive, and energy-efficient solutions must be developed^10,11^.

Recently, All-optical modulation has received considerable interest due to its ability to circumvent the speed and heat generation issues enforced by electro-optical modulators^12–14^. At the same time, all-optical signal processing is frequently viewed as a game-changing technology for the upcoming generation of computing and communication devices especially with the introduction of new computational requirements like artificial intelligence^15–17^. Accordingly, nonlinear optics is frequently employed these days in a variety of photonic devices, for implementing all-optical data modulation^18,19^. In perspective of potential implementation, the performance of these all-optical modulators employing nonlinear optics depends on extinction ratio (expressed as the contrast between "ON" and "OFF" states) and device footprint^1,7^. However, many a times these device applications are restricted by weak light matter interaction, with many of the incorporated materials displaying incredibly low optical nonlinearity^20–23^. As a result, the device exhibits low extinction ratio, high power consumption and a large device footprint making on-chip integration difficult. Furthermore, most optical modulation materials and geometry are inconsistent with conventional complementary metal–oxide–semiconductor manufacturing techniques^24–28^.

Henceforth, material and device engineering can be done while taking into mind the CMOS compatible process to achieve the necessary performance metrics, i.e., high extinction ratio and small device footprint. Regarding tailoring the material properties for achieving efficient all-optical modulation, Epsilon-near-zero (ENZ) materials have awhile back received much interest, not just for their exciting linear properties, but mostly for their large optical nonlinearities^29–31^. Furthermore, a portion of ENZ materials, transparent conductive oxides (TCO’s), illustrate near-infrared resonance frequencies, suggesting the possibility of integrated telecom applications^29–34^. Indium tin oxide (ITO) is a potential TCO which has shown to undergo a refractive index change of order unity when a thin film is optically pumped^35–42^. ITO absorbs photons with energies less than the bandgap via carrier absorption in its conduction band. Photon absorption causes hot-electron redistribution near the Fermi surface, resulting in a change in the real and imaginary permittivity components, resulting in optical nonlinearity of ITO^30–32^.

Micro ring resonance-based modulators have emerged as a valuable innovation in device engineering, especially when it comes to shrinking the size of optical modulators while maintaining their efficiency. These modulators are built around a fundamental concept that enhances their performance: the utilization of micro-ring resonators^43–47^. Unlike traditional single-pass devices, where light traverses the waveguide once, micro rings create a closed-loop optical path, allowing the light to circulate within the ring multiple times. This cyclic pathway introduces a crucial element into the design: each time the light circulates through the ring, it interacts with the surrounding material. This repetitive interaction significantly strengthens the coupling between the light and the material, leading to enhanced light-matter interaction. This confinement of light within the micro ring allows it to interact with the material over a longer path, amplifying the opportunities for nonlinear optical effects and modulation^48–50^.

The primary highlight of the present invention is all-optical modulation, which is thought to be of greater interest due to its ability to avoid the speed and heat generation issues imposed by electro-optical modulators. We propose an approach for all-optical modulation in an ITO based vertically coupled ring resonator employing the enhanced nonlinear response across the ENZ state of ITO in near infrared wavelength. The novel design of ITO based vertically coupled ring resonator is incorporated in the proposed work to circumvent the scattering losses and to enhance the light matter interaction enhancing sensitivity, non-linear effects, and compactness. The elimination of the need for fine gap etching between the ring and bus waveguide by employing a vertically coupled design is a significant departure from conventional approaches. The proposed structure allows the efficient coupling of high intensity pump wavelength into the ITO based micro-ring resonator, and thus this optical pumping in ITO will lead to electron heating accompanied by higher value of nonlinear optical parameters resulting in the change of effective mass because of the non-parabolic electron dispersion. This effect further leads to a change in overall refractive index of ITO in the unity order resulting in the modification of complex effective index of the propagating optical beam at 1550 nm. Efficient all-optical modulation is experimentally realized by optical pumping the ITO based vertically coupled ring resonator and a high extinction ratio of 18 dB for 30 µm long device is reported. The manuscript comprehensively addresses several key aspects. It begins by elucidating the design of the proposed device, including its modelling, which considers fabrication tolerances. The modelling of the vertically coupled ring resonator is done employing Lumerical Mode and Finite Difference Time Domain (FDTD) Solutions and Device. Subsequently, the paper delves into the material processing phase and discusses the practical realization of the nonlinear optical constants of ITO. An optimization of the ITO’s carrier concentration at different oxygen partial pressure is realized to attain epsilon near zero state. The non-linear optical constants are also measured by incorporating Z-scan measurements. Then the fabrication of the proposed structure is discussed which done employing ion beam sputtering, mask less lithography, and etching. And finally comprehensive discussions on all-optical measurements is done. Furthermore, it is worth mentioning here that this concept is first of its kind, incorporating vertically coupled ring resonator for realizing all-optical modulation with a simple fabrication process. We have not incorporated any of the conventional methods or device design to attain efficient all-optical modulation. The previously reported optical modulators are solely based on electro-optic effect and thermo-optic effect. The device design of such optical modulators usually incorporated p-i-n, pn junction geometry in Si or metal–oxide–semiconductor structure. The MOS type optical modulator usually leads to high propagation losses whereas the p-i-n geometry leads to slower device speed. The reported mechanism is of interest for the future high-speed data communication systems.

## Proposed device design and analysis

**Figure 1:**
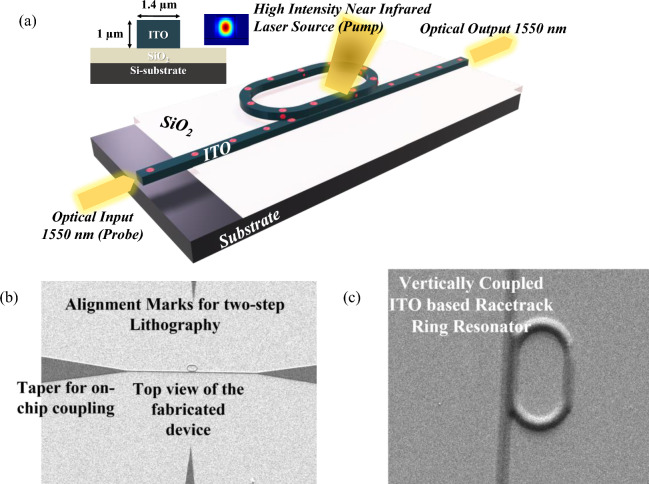
(**a**) Schematic representation of an engineered ITO based vertically coupled ring resonators in all-pass composition with a ITO bus and ring of width w = 1.4 µm and thickness (ITO) = 1 µm. The ITO racetrack ring resonator has a radius of 3.6 µm with a straight coupling region of 2 µm. The proposed vertically coupled racetrack ring resonator has zero coupling gap and length. The inset-on left shows the optical field propagation through ring resonator at on and off resonance. The inset on right exhibits the two-dimensional schematic of the device with optical mode. (**b**) The scanning-electron-microscope outlook of the top-view of the fabricated ITO based vertically coupled racetrack ring resonator along with the on-chip input and output grating coupler. (**c**) The zoomed scanning-electron microscopic image of top-view of the vertically coupled ITO based racetrack ring resonator.

A n-type ITO based vertically coupled ring resonator is designed to operate at 1550 nm. In the proposed micro-ring resonator, light is coupled into the ring waveguide via evanescent field coupling from the bus waveguide with a vertical coupled configuration. The structure is built on a silicon-on-insulator (SOI) substrate with 220 nm device layer thickness, 2 μm buried oxide (BOX) layer thickness, and 700 μm handle layer thickness. In order to avoid light leakage in Si caused by the tendency of light to propagate in materials with higher refractive indices, 500 nm of SiO_2_ is positioned above the SOI substrate. Figure 1 depicts the ITO based vertically coupled ring resonator, where ITO’s bus waveguide is placed above the silicon dioxide (SiO_2_) layer. The ITO’s ring waveguide is placed just above the bus waveguide. SiO_2_ has actually been placed next to the bus waveguide to support the ring waveguide. For attaining the proper working of micro ring resonator, we first design the ITO’s bus waveguide dimension such that only a single mode can be supported. The ring-resonator and bus waveguide are developed in an all-pass racetrack configuration with a ITO bus and ring of width w = 1.4 µm and thickness (t_ITO_) = 1 µm. The ITO racetrack ring resonator has a radius of 3.6 µm with a straight coupling region (Coupling Length) of 2 µm. The proposed vertically coupled racetrack ring resonator has zero coupling gap.

**Figure 2:**
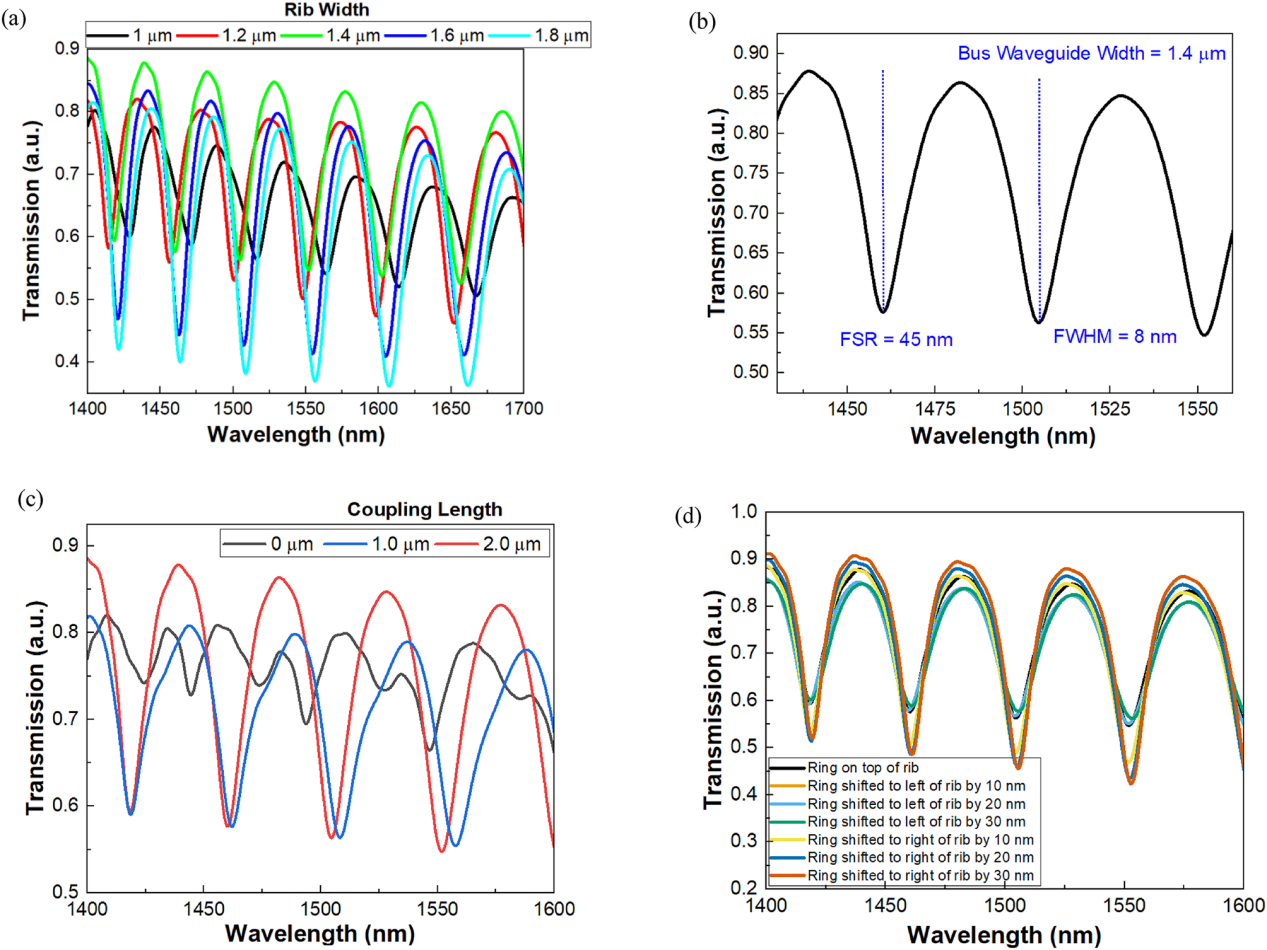
(**a**) exhibits the optical spectrum at the through port of the ring structure at different ITO’s rib width varying from 1 to 1.8 µm with a rib thickness of 1 µm. (**b**) exhibits the highest transmission attained at the rib width of 1.4 µm. The transmission characteristics also show the Fullwidth-half-maximum (FWHM) and Full Spectral Range (FSR) of the proposed vertically coupled racetrack ring resonator. (**c**) exhibits the optical transmission for different coupling length at the rib width of 1.4 µm. (**d**) exhibits the optical transmission spectrum considering the lateral offset in the ring waveguide with respect to bus waveguide.

The modal and field-propagation assessments of the ITO based ring-resonator are performed using the Lumerical Finite Finite difference-eigenmode and Finite-difference time-domain (FDTD) solutions, with the absolutely matched layer boundary settings. The proposed waveguide supports the fundamental TE-mode across near infrared wavelength. The structure parameters are thoroughly optimized to have a good evanescent coupling between bus and ring waveguide and to assure the on and off resonance states. For vertically coupled ring resonators, the lateral offset between the bus and the ring waveguides play an important role in determining the efficiency of the evanescent light coupling process. The inset in Fig. 1 exhibits the two-dimensional mode profile in the ITO’s bus waveguide and optical field propagation through vertically coupled ITO based racetrack ring resonator at on and off resonance respectively. The optical beam of resonant wavelength is efficiently coupled to the ring by optimizing the lateral offset. The optical waves in the ring create a round trip phase shift with an intensity equal to an integer multiplied by 2π, after which the waves constructively interfere, resulting in the resonance state. Figure 2(a) exhibits the optical spectrum at the through port of the ring structure at different ITO’s rib width varying from 1 to 1.8 µm with a rib thickness of 1 µm. The transmission exhibits resonant behavior at multiple wavelengths ranging from 1.4 to 1.8 µm. The highest transmission is attained at the rib width of 1.4 µm. Figure 2(b) depicts the transmission characteristics where the Fullwidth-half-maximum (FWHM) of 8 nm and Full Spectral Range (FSR) of 45 nm is determined and finesse calculated is FSR/FWHM = 5.625. To calculate the group delay in the ring structure, the time monitor is placed at the through port, the field data extracted from a time monitor and a Fourier transform is performed. The phase information is extracted after the Fourier transform and subsequently the derivative is taken to generate the group delay. The group delay of 48 psec is attained for 30 μm long device. Because light passes through the waveguide multiple times, the large group delay is achieved, allowing for better interaction than a conventional rib waveguide. The ring, which is connected to a bus waveguide, traps light independently, increasing the optical path length and light matter interaction.

In the proposed structure, we have also carried out the fabrication tolerance analysis with respect to the coupling length and gap of the racetrack resonator. The coupling length directly affects the resonance wavelength of the ring resonator. A properly chosen coupling length can enhance the interaction between light and matter within the ring resonator. This increased interaction is beneficial for various optical signal processing applications, including modulation, switching, and sensing. Figure 2(c) depicts the optical transmission spectrum for different coupling length for the rib width of 1.4 µm. The highest transmission has been attained for the coupling length of 2 µm. The lateral offset between the bus and ring plays a critical role in determining the efficient light guidance in the structure. Parallelly, the lateral offset between bus and ring indeed enacts in the fabrication process as well. Thus, the fabrication tolerance of the device with respect to the lateral offset has been computationally checked. Figure 2(d) exhibits the optical transmission spectrum considering the lateral offset in the ring waveguide with respect to bus waveguide. The transmission spectrum shows that the device design efficiently couples the light from bus to ring with an offset of ± 30 nm.

## Material analysis and calculation of nonlinear optical constants of ITO

The creation of materials whose refractive index can be significantly altered by a low-power optical field has long been an aim in nonlinear optics. These materials should ideally work with current CMOS fabrication processes. Amid of various materials, ITO is CMOS compatible and has ability to undergo a refractive index change of order unity when a thin film is optically pumped. Simple logic reveals that for a change in permittivity $\Delta \varepsilon$, the change in refractive index $\Delta n$ of ITO is denoted as $\Delta n=\Delta \varepsilon /(2\sqrt{\varepsilon }$). As the permittivity decreases, we observe that this change increases, indicating that ITO’s epsilon near-zero (ENZ) frequencies should result in significant nonlinear optical characteristics.

In this section, we've examined how the ENZ condition at telecommunication wavelengths influences ITO's improved optical nonlinearity. First, we experimentally confirmed the ENZ state of ITO. In order to determine the permittivity of ITO at 1550 nm wavelength for various ITO’s carrier density, we deposited ITO on thermally oxidized SOI wafer as well as quartz substate using ion beam sputtering and then conducted hall effect and ellipsometry measurements.

**Figure 3:**
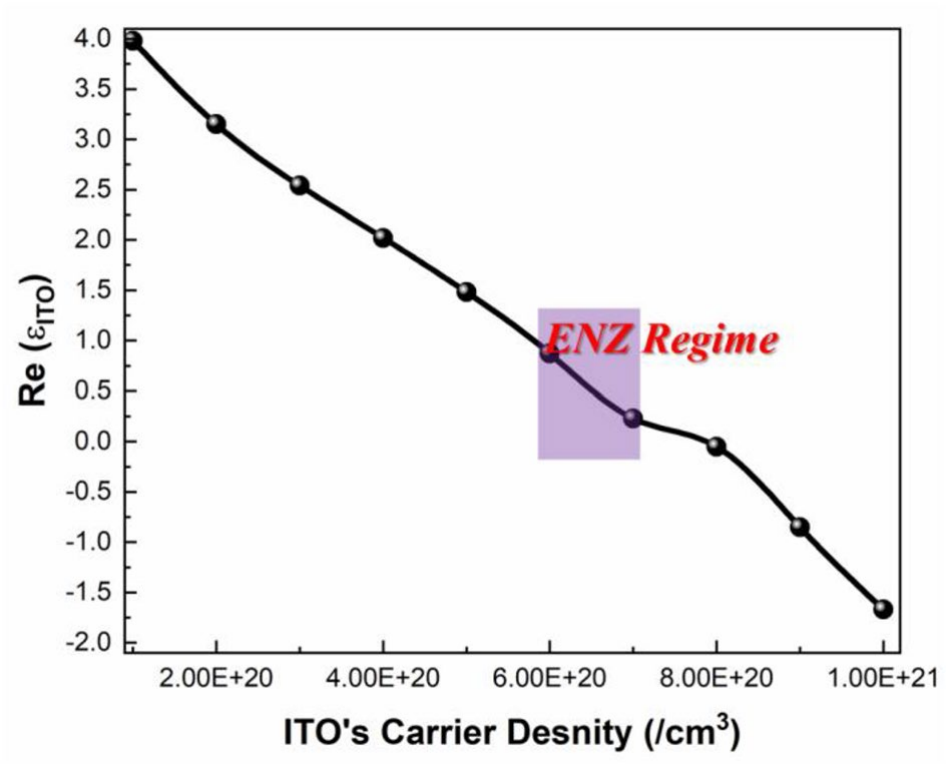
ITO optical attribute i.e., Plot of the real part of ITO permittivity as a function of ITO’s carrier density at $\lambda =1550\,{\mathrm{nm}}$ with shaded region exhibiting epsilon near zero state in ITO.

A commercially available 99.99% pure ITO’s ceramic target is used to carry out deposition on respective substrates by varying the oxygen partial pressure from 0.2 sccm to 0.7 sccm and Argon partial pressure is kept constant at 40 sccm. The background and working pressures are set at 5.7E−7 mbar and 7.5E−3 mbar respectively, while the ion-beam voltage and power are fixed at 180 V and 40 W, respectively. Deposition happens at a speed of 0.5 Å/s. After the deposition, on every quartz substrate the hall-effect measurement has been performed to estimate the carrier density of ITO. The ITO deposited at the lower oxygen flow rate of 0.2 sccm exhibited the metallic behaviour with a carrier density of about 1.178 × 10^21^/cm^3^ and the ITO deposited at the higher oxygen flow rate of 0.7 sccm exhibited the carrier density of 4.4 × 10^20^/cm^3^. The optical constants of the deposited ITO sample are measured using a J. A. Woollam M-2000 DI spectroscopic ellipsometer because it can characterize thin films quickly and accurately across a wide spectroscopic range. For three different angles of 65°, 70°, and 75°, VASE data are extracted. The VASE data is fitted with an ITO general-oscillator model, and the associated optical attribute of ITO i.e. Real part of the permittivity are discovered. The fit is strengthened by a mean-square error of 4.33, the originality of the calculated thickness parameter, thickness matching from deposition, and these factors combined. Figure 3 depicts the real part of permittivity with respect to different carrier density of ITO at a wavelength of 1550 nm. At the carrier density between 6 × 10^20^/cm^3^ to 7 × 10^20^/cm^3^, the real component of permittivity rapidly declines and comes close to zero. The ENZ state is identified as the wavelength when the permittivity is very close to zero. The deposited ITO layer has a real permittivity of ITO is 0.6 at 1550 nm at the carrier density of 6.5 × 10^20^/cm^3^, as shown in Fig. 3. Thus, ENZ state has been attained at the operating wavelength.

**Figure 4:**
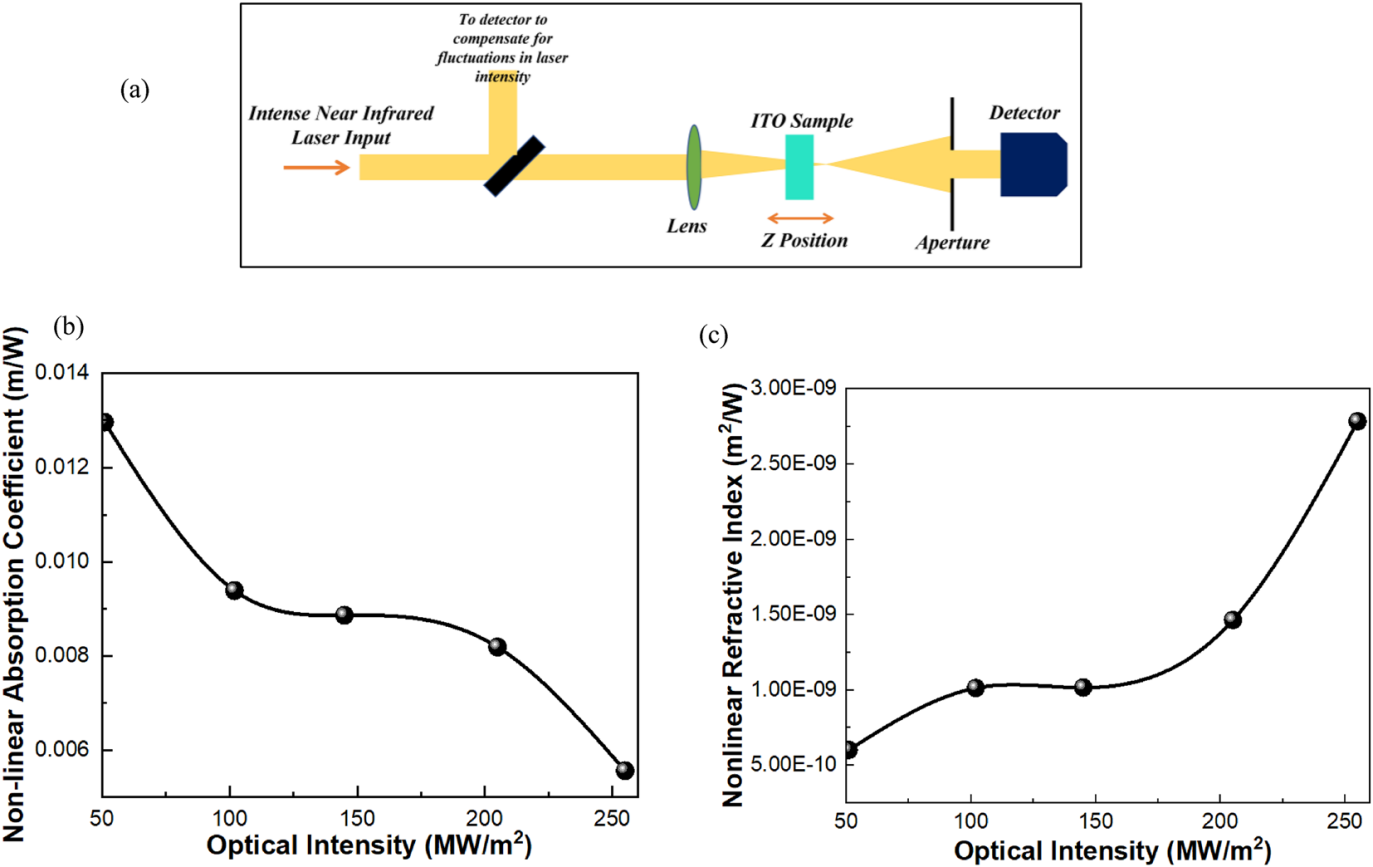
(**a**) Schematic exhibits the Z-scan setup which consists of tunable intense near infrared laser source, beam expander assembly, sample holder, lens, photodetector, and a digital power meter. (**b**) Plot exhibits the nonlinear absorption coefficient of ITO at different laser intensities. (**c**) Plot exhibits the nonlinear absorption coefficient of ITO at different laser intensities.

After attaining the ENZ state, Z-scan measurements have been done on the ITO sample exhibiting ENZ state i.e. the one having the carrier density of 6.5 × 10^20^/cm^3^ to calculate the nonlinear optical constants. To determine the nonlinear optical parameters, Z-scan proves to be the most versatile technique. From Z-scan technique we can examine the nonlinear absorption coefficient as well as nonlinear refractive index. Figure 4(a) exhibits the Z-scan setup which consists of tunable intense near infrared laser source, beam expander assembly, sample holder, lens, photodetector, and a digital power meter. In Z-scan experiment, a gaussian laser beam in a tight focused geometry is used to measure the transmittance of the deposited ITO sample as a function of the sample position (z) measured with respect to focal plane. Z-scan measurement has been performed in two ways viz open aperture and closed aperture z-scan measurement. In open aperture measurement, all the transmitted light is collected to the detector using a convex lens of large aperture to measure the nonlinear absorption coefficient of ITO. The measured normalized transmittance T (z) as a function of distance from the beam focus is then fitted using nonlinear curve fitting. For curve fitting, we define the normalized transmittance in open aperture by the equation:$T\left(z\right)={\sum }_{m=0}^{\infty }\frac{{[-{q}_{0}\left(z\right)]}^{m}}{{(m+1)}^\frac{3}{2}}$ ; ${q}_{0}\left(z\right)<1$. Here z is sample position, m is integer, ${q}_{0}\left(z\right)$ is the fitting parameter denoted as.

${q}_{0}\left(z\right)=\beta {I}_{0}{L}_{eff}/(1+\frac{{z}^{2}}{{z}_{R}^{2}})$ and ${L}_{eff}=\left[\frac{1-{\exp}\left(-{\alpha }_{0}L\right)}{{\alpha }_{0}}\right].$ Here β, I_0_, L_eff_ being the nonlinear absorption coefficient, laser light radiance at focal point and effective length of the ITO deposited sample respectively. Knowing the value of q_0_ from the theoretical fit of the experimental data, we have calculated the nonlinear absorption coefficient at different intensities of light. Figure 4(b) exhibits the nonlinear absorption coefficient of ITO at different laser intensities. With the increasing laser intensity there is decrease in the nonlinear absorption coefficient of ITO.

In closed aperture measurement, only the on-axis light is allowed to enter the detector to determine the nonlinear refractive index of deposited ITO. We have measured the normalized transmittance as a function of distance near the focal region of the lens. Under close aperture Z-scan condition, the normalized transmittance as a function of distance is denoted as: $T\left(x, \Delta {\varnothing }_{0}\right)=\left(1+\frac{4\Delta {\varnothing }_{0}x}{\left({x}^{2}+1\right)\left({x}^{2}+9\right)}\right)$. Here x is the dimensionless sample position given by $z/{z}_{R}$, $\Delta {\varnothing }_{0}$ represents the induced phase shift denoted as $\Delta {\varnothing }_{0}={n}_{2}{I}_{0}{L}_{eff}$, where n_2_ is the nonlinear refractive index and k is propagation vector. Using the above equation in nonlinear curve fitting $\Delta {\varnothing }_{0}$ is theoretically obtained. Using $\Delta {\varnothing }_{0}$, the nonlinear refractive index n_2_ of deposited ITO sample is calculated at different laser intensities. Figure 4(c) exhibits the nonlinear absorption coefficient of ITO at different laser intensities. With the increasing laser intensity there is increase in the nonlinear refractive index of ITO.

The main cause of the observed nonlinearity in Fig. 4(b) and (c) is the laser-induced electron heating, which changes the conduction-band electrons' energy distribution. Two things set ITO's hot-electron-induced optical nonlinearity at ENZ wavelengths apart from noble metals under infrared irradiation. First, as previously stated, the ENZ region always has a bigger nonlinear change in refractive index than non-ENZ regions for a given change in permittivity. Second, compared to a noble metal like gold, the free-electron heat capacity of ITO is lower. As a result, the difference in ITO's refractive index and the increase in the free-electron temperature over the Fermi temperature are substantially bigger. The nonlinear response of ITO at ENZ wavelengths can result in variations in its refractive index that are greater than the linear refractive index for sufficiently high optical intensities. This alteration of ITO’s refractive via intense optical pumping opens gateways for the realization of efficient all-optical modulation in vertically coupled ITO based racetrack ring resonator.

## Device fabrication

The fabrication of the vertically coupled ITO based racetrack ring resonator started with a conventional cleaning of the SOI wafer. After the wafer cleaning, ion beam sputtering is employed for the deposition of 500 nm of SiO_2_ using a commercially available SiO_2_ target. The deposition is carried out at a Argon partial pressure of 30 sccm with ion beam voltage of 100 V and power 20 W. Followed by this, a 1 µm thick ITO is deposited at oxygen partial pressure of 1 sccm and Argon partial pressure of 30 sccm. The ITO’s deposition parameters remain the same as mentioned in Section III. Further for the patterning of rib waveguide in ITO along with the input and output coupler, a positive photoresist S1813 is spin coated at 4000 rpm for 45 s followed by baking of photoresist at 115℃ for 60 s. Then PICOMASTER maskless lithography is employed for exposing the wafer with the desired mask of bus waveguide (width 1.4 µm) with proper alignment marks and couplers. The photolithography is performed with a dose of 100 µC/cm^2^, accelerating voltage of 10 kV and spot size of 300 nm. After the exposure the wafer is developed in 100% concentrated solution for 40 s. After development sample is post baked at 110℃ for 30 min. Then ITO is wet etched HCl Aqueous solution (4:1 HCl to DI water volumetric ratio, where HCl is the standard 37% HCl Solution) to attain the desired depth of 1 µm in bus waveguide. The etch rate for the wet etching of ITO is calculated as ≈ 48 nm/min. Moving ahead SiO_2_ is deposited on the sideways of bus waveguide to give the height to the ring waveguide utilizing a metal mask. Again, the same ITO deposition is carried out followed by photolithography to transfer the pattern of racetrack ring waveguide (over the bus waveguide with precise alignment marks. desired dimension of ring waveguide (radius of 3.6 µm with a straight coupling region of 2 µm). And again, the ITO’ wet etching has been carried out to attain the desired depth in ring waveguide. Figure 1(b) exhibits the scanning electron microscopic image of the fabricated vertically coupled ring resonator with alignment marks.

## All optical measurements

**Figure 5:**
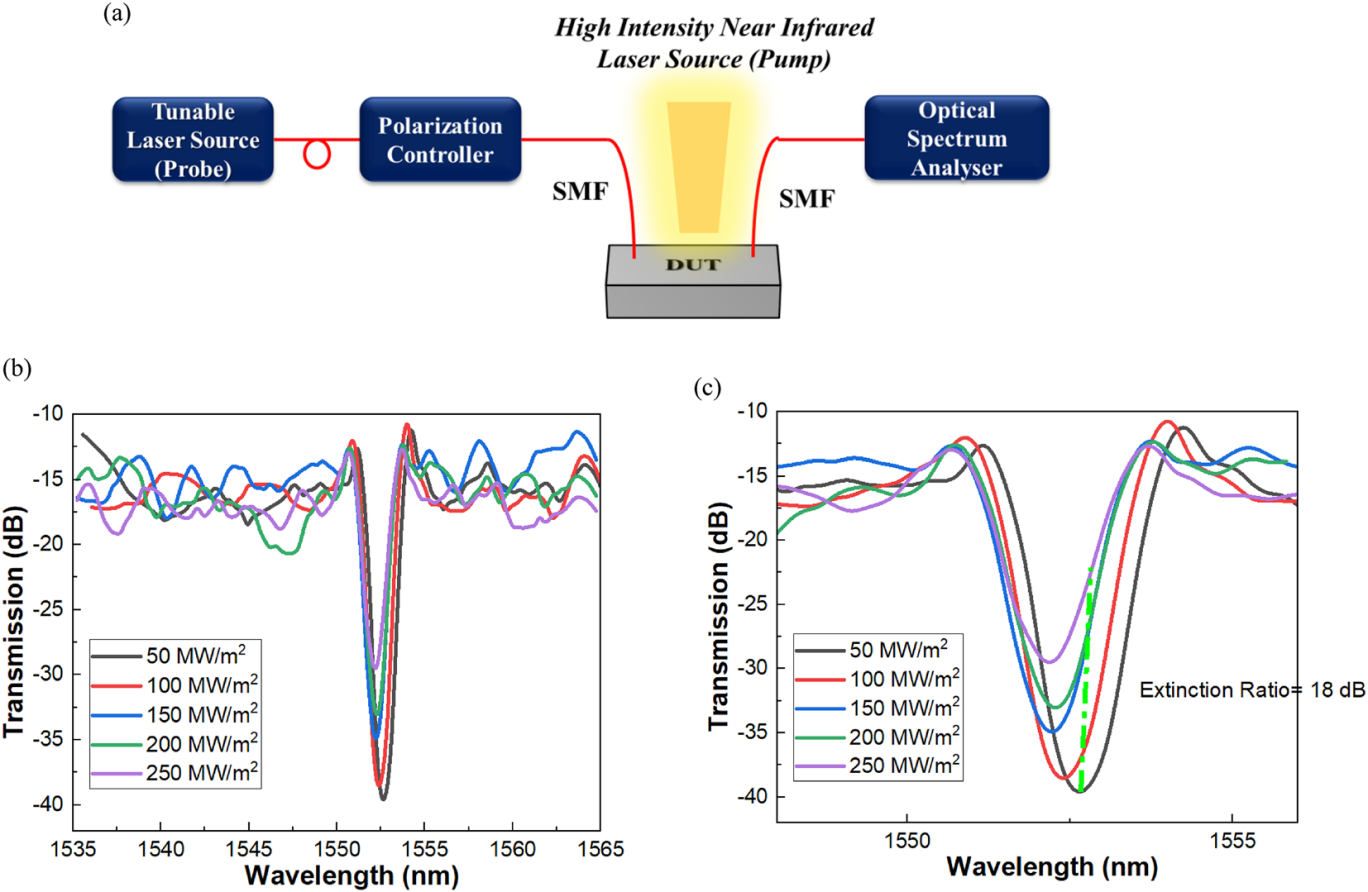
(**a**) displays a schematic illustration of the all-optical measuring system. The system includes a polarization controller, a microscope, a tunable intense near-infrared laser source as a pump, a high-resolution optical spectrum analyzer, and a laser source with a wavelength of 1527–1566 nm as a probe. (**b**) Plot exhibits the measured change in the transmission spectrum at different laser intensities. (**c**) Plot depicts an extinction ratio of 18 dB for 30 µm long device.

Figure 5 displays a schematic illustration of the all-optical measuring system (a). The system includes a polarization controller, a microscope, a tunable intense near-infrared laser source as a pump, a high-resolution optical spectrum analyzer, and a laser source with a wavelength of 1527–1566 nm as a probe. The optical mode at the input grating coupler of the device under test, an ITO-based vertically coupled racetrack ring resonator, is excited by the fiber-optic output from the probe laser source. A single-mode lensed fibre at the opposite end of the device is used to gather the transmitted light. The input and output single-mode lensed fibres are oriented at a 10° angle with respect to the surface normal of the test device in order to maximize the coupled power. In order to increase the nonlinearity of the material, an intense tunable infrared laser pump is projected from the top over the ITO's ring resonator. ITO's nonlinear optical constants will change when the pump wavelength is applied from above at various intensities because laser-induced electron heating alters the energy distribution of the conduction-band electrons. Due to this modification in ITO’s refractive index, the complex effective index of the propagating optical beam at 1552 nm will be modulated.

Figure 5(b) exhibits the measured change in the transmission spectrum at different laser intensities. With the increasing laser intensities there is an increase in the transmission through the ring resonator at 1552 nm. From Fig. 5(c) we have observed an extinction ratio of 18 dB for 30 µm long device. So, in the suggested all-optical ITO based ring resonator, a high extinction ratio with a small footprint has been achieved.

Since ITO's nonlinear absorption coefficient is decreasing and its nonlinear refractive index is rising with increasing optical intensity, the rise in transmission with increasing laser intensity is explained by both of these phenomena. As a result, the described method for achieving effective all-optical modulation in a vertically coupled ITO-based racetrack resonator constitutes a workable substitute for alternative, less effective Si and LiNbO3 state-of-the-art optical modulators. We have not incorporated any of the conventional methods or device design to attain efficient all-optical modulation. The previously reported optical modulators are solely based on electro-optic effect and thermo-optic effect. The device design of such optical modulators usually incorporated p-i-n, pn junction geometry in Si or metal–oxide–semiconductor structure. The MOS type optical modulator usually leads to slower device speed.

Table 1 presents a comparative analysis of the proposed all-optical modulator in terms of the extinction ratio (ER) and the physical size of the device, considering other works in the field of optical modulation. Our current work is juxtaposed with existing devices, including the ITO-based MOS modulator, the conventional Si-ITO heterojunction-based intensity modulator.

**Table 1:** Comparison of the performance of the proposed device with state-of-the-art devices in terms of ER and linear device footprint.

Material	Device Configuration	Linear Footprint (µm)	Extinction ratio ER (dB)
ITO^39^	Multiple Si-ITO heterojunctions based subwavelength grating in a rib waveguide	80	24
Si^41^	Hybrid plasmonic waveguide	1	1
ITO^42^	Hybrid waveguide based on Si-ITO heterojunction	1000	7
Graphene^51^	Stereo Graphene Microfiber Structure	Not Available	7.5
Graphene^52^	Plasmon Enhanced Graphene All-Optical Modulator	12	3.5
ITO^53^	Grating based All-Optical Modulator	Not Available	6.45
Lithium Niobate (LiNbO_3_)^54^	Mach–Zehnder modulator	7000	8
Si^55^	Mach–Zehnder modulator	3000	3
ENZ-Si^56^	ITO-HfO_2_-silicon metal–oxide–semiconductor (MOS) capacitor with ITO	1	0.1
Graphene^57^	Monolayer graphene on Si bus waveguide	1	0.1
Graphene^58^	Double-layer graphene on Si bus waveguide	1	0.16
ITO [This Work]	Vertically Coupled Micro-Ring Resonator	30	18

Notably, our all-optical modulation approach showcases competitive performance in both extinction ratio and device footprint. Consequently, our work introduces an appealing alternative to state-of-the-art optical modulators. It's important to highlight that our approach employs a novel ring resonator design to achieve resonance-enhanced optical confinement without the need for metallic components or plasmonic interactions.

In contrast, many photonic designs often incorporate techniques like surface plasmon excitation or ring resonators to enhance light-matter interactions for efficient optical systems and devices. While surface plasmon polaritons (SPP) can significantly enhance the optical field at the metal–dielectric interface, they suffer from limited propagation distances, which restrict their practical usability. Various strategies have been developed to extend SPP propagation, but this often comes at the cost of reduced field enhancement, resulting in a trade-off between light-matter interaction and optical losses. Henceforth, our novel ring resonator design for all-optical modulation leverages the nonlinear properties of ITO, enabling effective light-matter interaction without the need for metals or incurring optical losses and simplifies the fabrication process, making it more cost-effective and accessible. This approach holds promise for a wide range of high-speed applications.

## Conclusions

Efficient all-optical modulation is experimentally realized by optical pumping the ITO based vertically coupled ring resonator and a high extinction ratio of 18 dB for 30 µm long device is reported. This is an innovative approach for attaining all-optical modulation by employing the enhanced nonlinear response across the ENZ state of ITO in near infrared wavelength. The vertically coupled ring resonator is incorporated in the proposed work to circumvent the scattering losses and to enhance the light matter interaction. To achieve epsilon near zero, the ITO carrier concentration is optimized at different oxygen partial pressures. Detailed material engineering has been carried out in order to explore material whose refractive index can be significantly altered by a low-power optical field, which has been a goal in nonlinear optics. It is worth mentioning here that this concept is the first of its kind, incorporating vertically coupled ring resonator for realizing all-optical modulation with a simple fabrication process. The proposed technique holds a great promise for the development of high-speed data communication systems in the future. The device and material engineering used in this study places a strong emphasis on the creation and testing of the photonic devices, which offers significant benefits for the Internet of the Things due to their power efficiency, low cost, increased intelligence, and smaller size. One-of-a-kind optical modulation is made possible by the employed vertically coupled ring resonator using the ENZ state of ITO. In conclusion, all-optical modulation has enormous potential for the development of computing and communication technology, especially for addressing the computational challenges offered by artificial intelligence. It is a game-changing technology with the potential for ultra-high-speed processing, increased bandwidth and capacity, improved energy efficiency, and the ability to enable new computing paradigms that could revolutionize a variety of industries and applications while enhancing the capabilities of AI systems and making it easier to realize complex computational tasks.

## Data Availability

All data generated or analyzed during this study are included in this published article [and its supplementary information files].
